# Inhaled Anesthetics: Beyond the Operating Room

**DOI:** 10.3390/jcm13247513

**Published:** 2024-12-10

**Authors:** Dana Darwish, Pooja Kumar, Khushi Urs, Siddharth Dave

**Affiliations:** 1 Department of Anesthesiology, University of Texas Southwestern Medical Center, Dallas, TX 75390, USA; 2 School of Medicine, University of Texas Southwestern Medical Center, Dallas, TX 75390, USA; 3 University of Texas Southwestern Medical Center, Dallas, TX 75390, USA

**Keywords:** inhaled anesthetics, cerebral ischemia, status epilepticus, status asthmaticus, myocardial ischemia

## Abstract

The development of inhaled anesthetics (IAs) has a rich history dating back many centuries. In modern times they have played a pivotal role in anesthesia and critical care by allowing deep sedation during periods of critical illness and surgery. In addition to their sedating effects, they have many systemic effects allowing for therapy beyond surgical anesthesia. In this narrative review we chronicle the evolution of IAs, from early volatile agents such as ether to the contemporary use of halogenated hydrocarbons. This is followed by a discussion of the mechanisms of action of these agents which primarily involve the modulation of lipid membrane properties and ion channel activity. IAs’ systemic effects are also examined, including their effects on the cardiovascular, respiratory, hepatic, renal and nervous systems. We discuss of the role of IAs in treating systemic disease processes including ischemic stroke, delayed cerebral ischemia, status epilepticus, status asthmaticus, myocardial ischemia, and intensive care sedation. We conclude with a review of the practical and logistical challenges of utilizing IAs outside the operating room as well as directions for future research. This review highlights the expanding clinical utility of IAs and their evolving role in the management of a diverse range of disease processes, offering new avenues for therapeutic exploration beyond anesthesia.

## 1. Introduction

The discovery and development of inhaled anesthetics (IAs) has played a pivotal role in medical and world history [1]. They have shaped events ranging from modern warfare to pandemics. The search for the “ideal” IA has focused on maximizing sedating effects and pharmacokinetic predictability while minimizing cardiovascular and other side effects [2]. Due to these favorable traits, nitrous oxide (N_2_O) and halogenated anesthetics, such as isoflurane, sevoflurane, and desflurane, have become essential components of modern anesthesia practice [3].

While there remain gaps in our knowledge, recent research has significantly advanced our understanding of the mechanisms of action of IAs [4]. And though the use of IAs has historically focused on blunting consciousness during painful procedures, newer understanding of how such agents work have led to their use outside the operating room to treat a variety of conditions including seizures, asthma, stroke, and myocardial ischemia [5]. In this review, we will explore the history and development of IAs, examine their mechanisms of action, and discuss their therapeutic role outside of surgical anesthesia.

## 2. Historical Overview

While the first use of the term “anesthetic” is ascribed to Oliver Wendell Holmes in a letter to American physician William Morton in 1847, descriptions of sensorium-altering substances given before surgery date back to ancient Egyptian and Indus valley civilizations [6,7]. Records from these periods show a variety of substances including hemp, poppy seeds, and mandrake root being administered during painful procedures. While many were consumed in raw form or as mixtures, there is reference to burning and inhaling of substances to induce hypnotic and paralytic effects.

**Figure 1:**
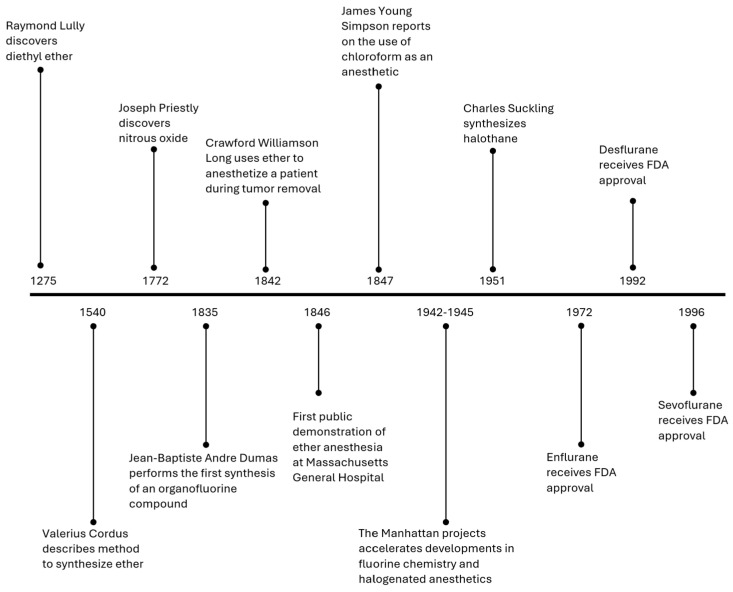
Important historical events in the development of inhaled anesthetics.

Diethyl ether is the first modern-era IA, though it was not initially used in this context [8] (Figure 1). It is believed to have been discovered by Raymond Lully, a 13th century Spanish alchemist, experimenting with combinations of sulfuric acid and wine [9,10]. In 1842, Crawford Williamson Long made this crucial connection between ether and surgical anesthesia, using a towel to administer ether during a neck tumor removal in Jefferson, Georgia. However, Long did not publish his findings until 1849, which cost him recognition as the discoverer of anesthesia [10].

In 1846, William Thomas Green Morton began experimenting with inhaled ether after learning of its analgesic properties on the skin. He successfully used it to anesthetize patients for dental procedures in his office. As his successes mounted, Morton sought to demonstrate ether publicly. He received an invitation to do so at the Bullfinch amphitheater of Massachusetts General Hospital, later known as the “Ether Dome”. On 16 October 1846, now celebrated as Ether Day, Morton administered surgical anesthesia to Edward Gilbert Abbott, a patient of surgeon John Collins Warren, for the removal of a jaw tumor [11]. On that day, Morton also demonstrated an inhaler he had developed which consisted of a large glass bulb containing an ether-soaked sponge and a spout for the patient to inhale through. Though Morton’s demonstration was groundbreaking and paved the way for “painless” surgery, it took years before anesthesia was universally adopted in medical practice.

In 1772, English chemist Joseph Priestly marked another turning point in the development of IA with the discovery of N_2_O [12]. Along with other scientists, including Joseph Black and Antoine Lavoisier, Priestly helped pioneer the field of “pneumatic medicine” by identifying and classifying atmospheric gases such as oxygen, hydrogen, and carbon dioxide [13]. In the early 1800s, self-proclaimed chemistry professor Gardner Quincy Colton hosted scientific exhibits and public demonstrations showcasing the effects of N_2_O [14]. In 1844, Horace Wells attended one of Colton’s demonstrations and became intrigued by the analgesic properties of this gas. Wells experimented with N_2_O for dental extractions, and in 1845 arranged a demonstration at Massachusetts General Hospital. However, the demonstration failed, likely due to inadequate delivery of the gas, discrediting N_2_O as a suitable anesthetic at that time [2].

During this time in Edinburgh, Scotland, obstetrician James Young Simpson began using ether for labor pain relief but was dissatisfied with its results [15]. Upon the suggestion of David Waldie, Simpson turned to chloroform as an alternative. In 1847, Simpson tested chloroform with friends at a dinner party, and their resounding success prompted him to submit a report on the use of chloroform to *The Lancet*. However, the prevailing belief at the time was that relieving labor pain was contrary to God’s will, and thus widespread adoption was slow. This debate subsided when Queen Victoria endorsed chloroform after John Snow successfully administered it during the deliveries of her own children [9]. In 1848, Snow established fundamental scientific principles of anesthesia by studying the relationship between vapor pressure, solubility, and anesthetic potency. He discovered the concept of minimum alveolar concentration (MAC), using animal models to determine the anesthetic concentration required to prevent movement in response to sharp stimuli [16]. His seminal work over the years also included other studies, such as the five stages of etherization and dose–response relationships for anesthetics [2].

The search for the ideal anesthetic continued into the second half of the 19th century, leading to the discovery of a second generation of IAs. Ethyl chloride, originally used as a topical anesthetic, was among the first of this era. In 1894, Swedish dentist H.J. Carlson sprayed ethyl chloride into a patient’s mouth to “freeze” a dental abscess, unexpectedly rendering the patient unconscious [17]. Later, in 1923, ethylene gas was explored as a potential anesthetic, but it proved unsuccessful due to the high concentrations required, which made it explosive and malodorous. In 1929, an accident led to the discovery of propylene as an anesthetic, but it was found to cause nausea and cardiac dysrhythmias after prolonged storage. Using a cylinder of deteriorated propylene, chemist George Lucas isolated and identified cyclopropane, which he later successfully tested on kittens [18]. In 1934, American scientist Ralph Waters from the University of Wisconsin conducted studies and reported on the clinical success of cyclopropane in humans. However, despite this productive era of discovery, most volatile anesthetics of the time were highly explosive. This concern led British anesthetists during World War II to adopt trichloroethylene, though its use was limited in the United States [9].

The first organofluorine compounds were synthesized by chemist Jean-Baptiste Dumas in 1835, though it was not until the 20th century that organofluorine chemistry was widely explored [19]. The development of atomic weapons programs in the mid-20th century brought important advances in fluoride chemistry. A total of 99.3% of all naturally occurring uranium exists as uranium-238, whereas uranium-235 is required as fissile material [20]. While separation is challenging due to their nearly identical physical properties, the discovery of gaseous uranium hexafluoride allowed centrifugal separation and enrichment of the 235 isotope to the 90% purity required for nuclear weapons [21]. Earl McBee of Purdue University pioneered the synthesis of this and other similar compounds through halogen exchange, resulting in the creation of one of the strongest bonds in organic chemistry, the carbon–fluorine bond [22].

While working on military applications, McBee, along with Vanderbilt pharmacologist Benjamin Robbins, received industry funding from Mallinckrodt to develop methods for synthesis of halogenated hydrocarbons [23]. Robbins’ recognition of the anesthetic potential of fluorinated hydrocarbons inspired work by others such as Ross Terrell and his colleagues, which yielded over 700 fluorinated compounds, including modern IAs such as enflurane, isoflurane, sevoflurane, and desflurane [24]. One of the first fluorinated anesthetics developed during this time was halothane, created in 1951 by British chemist Charles Suckling. Suckling offered halothane to prominent British anesthetist Michael Johnstone and it quickly became popular worldwide, gaining FDA approval in 1958 despite concerns about its association with hepatitis [25,26]. In 1958, methoxyflurane was the next to be discovered and remained popular for a decade, until concerns of dose-related nephrotoxicity arose with prolonged use [27]. These side effects fueled the ongoing search for the “perfect” volatile anesthetic with metabolic stability. An important advantage of fluorinated alkanes during this era was their significantly lower combustibility compared to earlier anesthetics like ether or cyclopropane.

Enflurane received FDA approval in 1972, but despite its potency and chemical stability, it was associated with significant complications related to reduced myocardial contractility and lowering of seizure threshold [28]. Isoflurane, after initial challenges with purification, gained wider acceptance in 1965 when Louise Speers and others published successful trials on its synthesis. Around this time, concerns about the side effects of existing IAs—halothane with hepatitis, isoflurane with coronary steal, and enflurane with cardiac and neurotoxicity—drove the development of desflurane and sevoflurane. Sevoflurane was first synthesized in the 1960s by healthcare company Baxter Travenol [29]. It was initially approved for human use in Japan in 1990, received FDA approval in 1996, and remains a commonly used anesthetic agent worldwide today.

Desflurane, also developed by Terrell in the 1960s presented unique challenges due to its high vapor pressure, leading to the invention of specialized heated vaporizers which allowed for greater consistency in anesthetic delivery [30]. Despite its pungent odor and tendency to cause airway irritation, the low solubility of desflurane allows for rapid induction and awakening.

Ongoing research into the narcotic effects of various gas mixtures during the mid-1900s led to the discovery of anesthetic properties of noble gases such as xenon. The first evidence of the anesthetic properties of xenon was published in 1946 by J.H Lawrence, who was able to demonstrate central nervous system (CNS) depression in mice following xenon inhalation [31].

In 1963, Edmond Eger described a technique for determining the MAC necessary to prevent movement in response to a surgical stimulus, and in 1965 showed that MAC was reproducible over a wide range of physiologic conditions and surgical durations [32,33] (Table 1). MAC continues to be the method by which IA dosing is described today.

## 3. Mechanisms of Action

Anesthesia is characterized by loss of consciousness, amnesia, analgesia, and muscle relaxation; these effects are mediated by various mechanisms through agent-specific molecular targets, which work together to produce the pleiotropic effects of IAs. IAs are low-molecular weight, non-polar, and hydrophobic in structure [34]. Claude Bernard first proposed a connection between IAs and protein function following his discovery that exposure to chloroform caused cessation of cytoplasmic movement within amoeba [35]. In 1901, Meyer and Overton published their findings that the potency of IAs was correlated with their solubility in non-polar solvents, implying that their anesthetic action results from interactions at lipophilic sites [36]. This led to the hypothesis that IAs produce anesthetic effects by affecting bulk lipid membrane properties [37]. However, this explanation has several limitations. The solubility–potency connection is only approximate, not all lipophilic molecules possess anesthetic action, and similar correlations as those found by Meyer and Overton have been replicated with both hydrophobic and amphipathic solvents [38]. Desflurane, enflurane, halothane, and isoflurane all possess a chiral carbon atom, with the (+) enantiomer showing greater activity at gamma-aminobutyric acid (GABA) and N-methyl-D-aspartate (NMDA) receptors [39]. While these effects are greater in vitro than in vivo, they indicate that lipid solubility alone does not account for the anesthetic potency of these agents.

## 4. Molecular Targets

**Figure 2:**
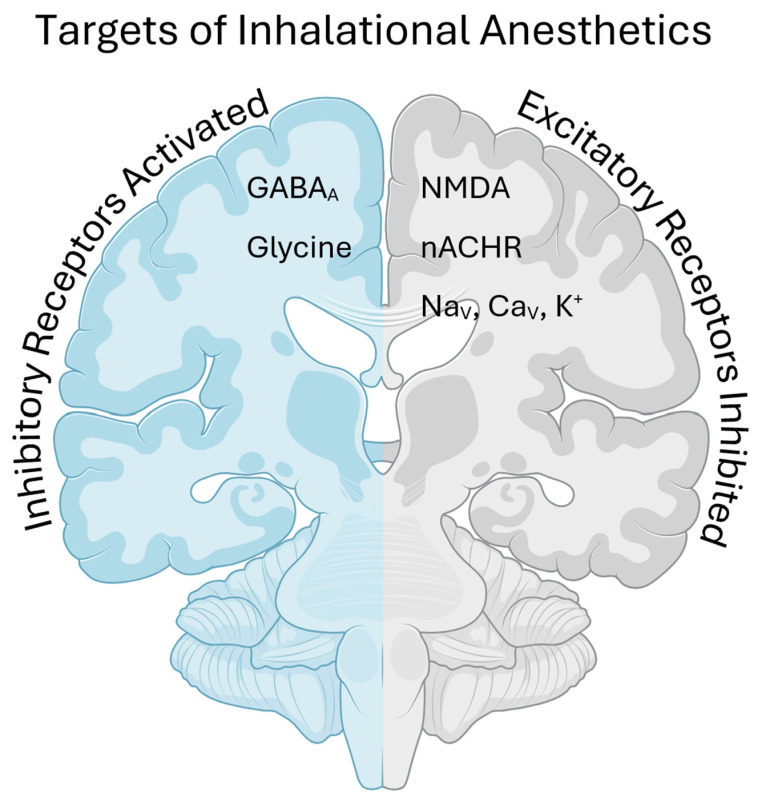
Inhaled anesthetics exert their sedating effects by activating inhibitory neurotransmitter receptors and inhibiting excitatory neurotransmitter receptors. (GABA, gamma amino butyric acid; NMDA, N-methyl D-aspartate; nACHR, nicotinic acetylcholine receptor; Na_v_, voltage-gated sodium channel; Ca_v_, voltage-gated calcium channel; K, potassium channel).

Four main criteria are used to identify molecular targets of anesthetic action: the anesthetic must reversibly and directly alter the target’s function at clinically relevant concentrations; the target must be expressed at specific anatomic locations that mediate the anesthetic’s functions; stereoselective effects on the target must be consistent between in vivo and in vitro experiments; and finally, pharmacologic or genetic disruptions that alter anesthetic sensitivity must abolish the anesthetic effect at that specific site [40]. Putative targets include inhibitory receptors such as GABA_A_ and glycine, excitatory receptors such as NMDA and nicotinic acetylcholine receptors (nACHR), potassium channels (K^+^), and voltage-gated sodium (Na_v_) and calcium (Ca_v_) channels (Figure 2).

## 5. Systemic Effects

**Figure 3:**
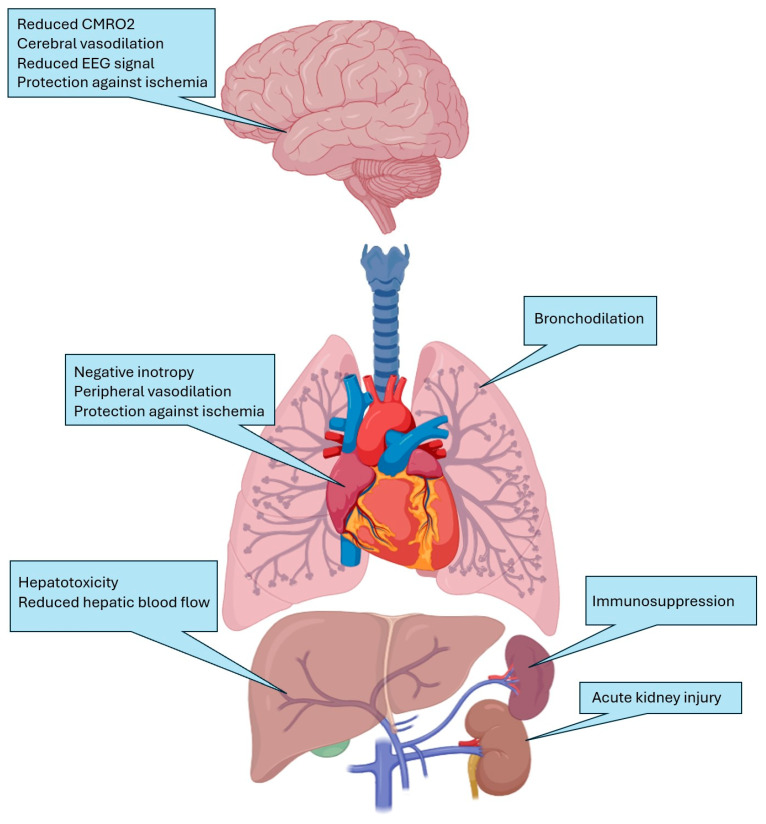
Systemic effects of inhaled anesthetics within and outside the central nervous system.

Due to the widespread distribution of target receptors, IAs have significant systemic effects (Figure 3). In addition to induction of anesthesia, IAs reduce cerebral oxygen consumption, promote cerebral vasodilation, and reduce frequency and amplitude of electroencephalograph (EEG) signals [41]. Inhibition of Ca_v_ and activation of GABA_A_ within airway smooth muscle leads to bronchodilation and reduced airway inflammation [42,43]. Inhibition of skeletal muscle nACHRs and enhancement of glycine receptors within the spinal cord can lead to dose-dependent muscle relaxation [44,45]. Additionally, activation of calcium-sensitive ryanodine receptors within muscle tissue can trigger malignant hyperthermia [46]. Reduced entry and altered handling of calcium within the sarcoplasmic reticulum of myocardial cells and vascular smooth muscle leads to direct negative inotropy and reduced systemic vascular resistance [47], though such complications are shared with many IV anesthetic agents. A randomized controlled trial (RCT) by Soukup et al. comparing intensive care unit (ICU) sedation with sevoflurane versus propofol did not show any significant differences in hemodynamics or rates of adverse events. [48]. Recent perioperative guidelines emphasize the importance of considering hemodynamic complications of anesthetic agents prior to administration, and similar care should be taken in non-operative settings, as well [49].

Halothane poses a notable risk of hepatotoxicity, and has been associated with fulminant hepatitis [50]. This hepatotoxicity is linked to the biotransformation of volatile agents by cytochrome P450 enzymes, leading to the production of trifluoroacetylated protein antigens. Halothane also significantly reduces hepatic blood flow, whereas isoflurane is considered safer in this context due to its ability to vasodilate the hepatic vasculature. IAs have been shown to inhibit neutrophil and lymphocyte activity via inhibition of Ca_V_, inflammatory cytokines, and superoxide production [51]. Methoxyflurane was withdrawn from the market following reports of refractory renal failure, secondary to high serum fluoride levels, while sevoflurane is associated with nephrotoxicity associated with the halide byproduct compound A, though clinical significance of this in humans has never been demonstrated [52,53]. Multiple case reports have demonstrated an association between prolonged use of sevoflurane and development of nephrogenic diabetes insipidus in ICU patients [54,55,56]. This is thought to be secondary to reduced aquaporin-2 channel activity, rather than from direct nephrotoxicity [57].

One of the most concerning effects of IAs is their potential to induce neurotoxic effects within the CNS. Multiple preclinical and clinical studies have implicated IAs in the development of postoperative cognitive dysfunction (POCD) in both animal models and humans. Impaired memory and spatial learning has been demonstrated in aged rats after exposure to sevoflurane, isoflurane, and N_2_O [58,59,60]. The data from human studies are less clear, both in the magnitude of cognitive effect of IAs and their difference compared to intravenous (IV) anesthetics. One retrospective, nested case–control study of 877 patients showed no significant association between IA exposure and cognitive dysfunction, while a prospective RCT of 921 patients showed reduced IA exposure is associated with reduced development of POCD [61,62]. Pooled analyses are similarly contradictory. In a meta-analysis by Pang et al., the incidence of POCD was markedly lower following propofol anesthesia than anesthesia with IAs, whereas a more recent meta-analysis by Huang et al. showed no difference in the incidence of POCD between inhaled and IV anesthetics [63,64].

While these studies examined POCD in adult patients, others have looked at the effects of IAs on children. Multiple landmark studies seem to indicate that IAs do not confer significant risk on cognitive development in pediatric populations. In the landmark GAS study, children who underwent hernia repair surgery during infancy using IAs did not display increased risk of cognitive dysfunction at 2 or 5 years of age compared to those who received regional anesthesia [65]. Similarly, the PANDA study showed that children exposed to either sevoflurane or isoflurane before age 3 showed no difference in cognitive development later in life compared to a matched, similarly-aged sibling cohort [66].

## 6. Inhaled Anesthetic Use Outside the OR

### 6.1. Ischemic Stroke

In patients with ischemic stroke, cerebral ischemia-reperfusion injury (IRI) is a major contributor to adverse outcomes. While the pathophysiology of cerebral ischemic injury is complex, evidence indicates that injury mechanisms involve oxidative stress, calcium overload, excitotoxicity, inflammatory cytokines, apoptosis, and increased permeability of the blood–brain barrier [67,68,69,70,71,72,73,74].

Inhalational anesthetics can provide neuroprotection against brain ischemia by three treatment patterns related to the timing of intervention relative to ischemia: preconditioning, proconditioning, and postconditioning. Preconditioning involves administration of low anesthetic doses prior to onset of ischemia [75,76]. Preconditioning improves tolerance to larger subsequent ischemic events through, as yet, incompletely understood humoral, cellular, and neural mechanisms [77]. Proconditioning involves administration of IA during periods of cerebral ischemia [78]. Postconditioning is the administration of IAs after an ischemic event has occurred [79,80].

Oxidative stress is a major driver of cerebral IRI [81,82]. In this setting, mitigation of free radical-induced damage in brain cells may be one of the protective mechanisms of sevoflurane against neurotoxicity. There is increasing interest in the use of sevoflurane as an anesthetic treatment for patients presenting with cerebral ischemia due to its ability to regulate antioxidant enzyme activity and inhibit oxidative stress [83,84].

Inflammatory mediators likely play a significant role in the development of ischemic brain injury. Nuclear factor kappa B (NF-κB) is produced early during neuronal injury and induces several downstream proinflammatory cytokines. In addition to inhibiting microglial cells, sevoflurane preconditioning may reduce the activation of NF-κB and downregulate the expression of NF-κB-dependent inflammatory factors such as cyclooxygenase-2 (COX-2), interleukin 6 (IL-6), interleukin-1α (IL-1α), interleukin-1β (IL-1β), tumor necrosis factor-α (TNF-α) and inducible nitric oxide synthase (iNOS), leading to reduced infarct volume and improved neurological function after ischemic brain injury [85]. This mechanism may not be universal across IAs, as using nonspecific nitric oxide synthase (NOS) inhibitors at 2 h after transient focal cerebral ischemia in rats did not alter the pretreatment effect of halothane [86].

NAD(P)H quinone oxidoreductase 1 (NQO1) can eliminate superoxide anion radicals and prevent the formation of reactive oxygen species [87]. Sevoflurane post treatment has been shown to activate the phosphatidylinositol 3-kinase/serine-threonine protein kinase (PI3-K/Akt) signaling pathway and upregulate the expression of NQO1 in ischemic brain tissue [88].

IRI can induce neuronal apoptosis through promotion of the E2F transcription factor 1 (E2F1)/enhancer of zeste homolog 2 (EZH2)/tissue inhibitor of metalloproteinases-2 (TIMP2) regulatory axis [89]. Sevoflurane reduces apoptosis of hippocampal neurons in rats with cerebral IRI by downregulation of E2F1 and activation of EZH2-mediated TIMP2 inhibition, thus playing a brain protective role in IRI [90].

Cerebral ischemia is associated with increased glutamate release and activation of AMPA and NMDA receptors, leading to excitotoxicity and neuronal injury [91,92]. Sevoflurane has been shown to reduce glutamate concentration in the rat cortex and hippocampus after global cerebral ischemia [93]. Administration of enflurane, halothane, sevoflurane, and isoflurane lead to reduced glutamate release in the brains of rats exposed to hypoxia and oxygen–glucose deprivation (OGD) [94,95,96]. IAs also directly antagonize AMPA and NMDA receptors. In in vivo studies of mouse cortex, administration of isoflurane attenuated excitotoxicity both during and after administration of AMPA [97]. Furthermore, halothane led to greater reduction of AMPA-mediated excitation compared with xenon, cyclopropane, enflurane, isoflurane and desflurane [98]. Isoflurane and sevoflurane antagonize NMDA-mediated excitotoxic signaling and NMDA-gated currents in hippocampal slices, cultured cortical neurons, and neuron–glial cell cultures [99]. Thus, attenuation of AMPA and NMDA signaling likely plays a significant role in the neuroprotective effects of IAs.

The concentration of Ca^2+^ in the brain plays an important role in the activation of NMDA receptors and opening of Ca_V_ [100]. Isoflurane preconditioning regulates the calcium-binding protein calmodulin and activates the MAPK extracellular regulated protein kinase pathway by increasing intracellular calcium in hippocampal neurons in a rat OGD model [101]. Similarly, preconditioning with isoflurane has been shown to maintain the calcium/calmodulin-dependent protein kinase II activity in a canine cardiac-arrest model [102]. In addition to the regulation on calcium channels and calcium-binding proteins, IA preconditioning also activates ATP-sensitive potassium channel (K^ATP^). K^ATP^ exists in sarcolemmal and mitochondrial forms within the brain and cerebral vasculature [103,104]. These channels, especially mitochondrial K^ATP^, play important roles in reducing or delaying cerebral cell death, as evidenced by reversal of neuroprotection by either glibenclamide or 5-hydroxydecanoic acid, two mitochondrial K^ATP^ blockers [105]. Isoflurane also activates K^ATP^ by activating adenosine A1 receptors and thus provides neuroprotection against focal cerebral ischemia [106].

While human data are insufficient to provide strict dosing guidelines, rat studies indicate that sevoflurane doses above 2% are sufficient to provide neuroprotection during and after cerebral ischemia [107,108]. Anesthesia-induced respiratory depression and hypotension may partially counteract some these preconditioning effects.

### 6.2. Delayed Cerebral Ischemia

The initial 24 to 48 h following aneurysmal subarachnoid hemorrhage (SAH) are referred to as the early brain-injury phase, characterized by disruption of the blood–brain barrier, neuronal cell death, neuroinflammation, cerebral edema, microthrombosis, abnormal depolarization events, and failure of cerebral autoregulation [109]. Accumulation of extravascular blood continues to aggravate these processes, resulting in high risk for vasospasm and delayed cerebral ischemia (DCI) for up to 10 days following injury [110]. Despite advances in critical care management of SAH and DCI, these conditions continue to confer high morbidity and mortality burdens [111].

Owing to their vasodilatory and neuroprotective properties, IAs have been proposed as a possible therapy for DCI. Several in vivo and animal studies have shown that sevoflurane pre-conditioning affords significant protection against SAH-induced DCI, including improved short-term neurological deficits through mechanisms that include upregulation of hypoxia inducible factor 1-α and increased expression of endothelial nitric oxide synthase [112,113,114,115,116]. A recent retrospective analysis by Athiraman et al. showed that the use of IAs for SAH patients undergoing aneurysm repair was associated with a lower incidence of angiographic vasospasm and DCI compared to those who received total intravenous anesthesia (TIVA) with propofol [117]. These results suggest that IAs may be preferable for this population. A large number of SAH patients are exposed to anesthetics very early on after ictus for aneurysm clipping/coiling or other ancillary procedures. It is essential to consider the advantages and disadvantages of different anesthetics and their potential impact on key outcomes such as angiographic vasospasm and DCI.

### 6.3. Status Epilepticus

Seizure and epilepsy carry a substantial risk for poor neurological outcomes. In a review of 596 cases of status epilepticus (SE) and super-refractory status epilepticus (SRSE), 13% of patients developed severe neurological deficit, 13% had mild neurological deficit, 4% had undefined deficit, and 35% died [118]. SRSE is associated with radiologic evidence of progressive brain atrophy [119]. Uncontrolled seizure activity upregulates NMDA receptors, resulting in calcium-mediated excitation, apoptosis, and necrosis [120]. In addition, GABA receptors are internalized from the extracellular membrane to the cytosol, reducing the effectiveness of GABA agonists such as benzodiazepines and barbiturates. Current seizure management guidelines advocate the use of benzodiazepines and anti-epileptic drugs as first-line therapies, with IV anesthetics as adjuncts [121].

IAs have been used occasionally for the treatment of SRSE when other treatments have failed [122]. While the exact mechanism by which these agents suppress seizures is not completely understood, they induce a rapid suppression of seizure activity under EEG monitoring. IAs can also provide cerebral protection via inhibition of NMDA receptor excitotoxicity, while also activating GABA_A_ receptors.

Not all modern IAs are equivalent, however. Sevoflurane has been demonstrated in both animal and human studies to be pro-epileptogenic [123]. The antiepileptic properties of isoflurane and desflurane seem to be superior, potentially via more potent NMDA inhibition [124]. Similarly, the safety of prolonged administration provides both isoflurane and desflurane with a potential advantage over sevoflurane and its predecessors, halothane and enflurane. In one case series of 14 infants treated for perinatal asphyxia encephalopathy, the use of 30% inhaled Xenon was associated with 100% seizure control in all patients [125].

The most common complication of IA treatment is hypotension, which may require the use of vasopressors, though there is also a concern for potential neurotoxicity. While evidence is limited, several case reports and case–control studies indicate that use of isoflurane for SRSE is associated with radiologic evidence of increased hippocampal damage [126,127].

In a systematic review by Zeiler et al., seizure control was achieved with IAs in 92.9% of adult patients and 94.4% of pediatric patients, with the majority responding almost immediately upon gas administration [128]. Desflurane was inferior to isoflurane in reaching seizure-control and burst-suppression thresholds. Hypotension was commonly described, but data were insufficient for statistically significant conclusions.

### 6.4. Status Asthmaticus

Asthma affects over 250 million people a year, with exacerbations responsible for 500,000 adult and child deaths per year [129]. Most asthma-related deaths are a result of status asthmaticus (SA), a life-threatening asthma exacerbation refractory to standard therapies [130]. This can lead to persistent hypoxemia and worsening respiratory failure requiring mechanical ventilation [131]. Mechanically ventilated ICU patients commonly receive sedation with IV anesthetic agents such as midazolam or propofol. Recent evidence suggests IA may offer benefit to patients being treated for SA.

When used in the ICU setting, sedation with sevoflurane is associated with a reduction in time to spontaneous breathing and extubation compared to that of propofol or midazolam [132]. And it has been shown to produce a bronchodilatory effect with minimal airway irritation, making it a popular choice for sedation in asthmatic patients undergoing surgery [133,134]. Sevoflurane-induced bronchodilation occurs through inhibition of Cl^−^ and K^+^ channels, resulting in bronchial smooth-muscle relaxation [135]. Rooke et al. described a reduced respiratory system resistance following treatment with IAs for five minutes in healthy volunteers [136]. The airway actions of IAs requires diffusion through the bronchial and tracheal lumen to reach the smooth muscle, and the speed of onset is dependent on the concentration gradient [137].

Other mechanisms by which IAs cause bronchodilation include inhibition of cholinergic transmission [138]. For example, sevoflurane-induced bronchodilation has been shown to occur through systemic absorption from the bronchial and pulmonary vessels to poorly ventilated lung regions, as well as reductions in vagally mediated parasympathetic activity [139,140,141]. Patients with SA tend to have poor responses to medical therapy and often require high ventilatory settings. This can lead to complications such as air trapping, barotrauma, and hypotension [142]. One retrospective case series of seven children admitted to the ICU with severe asthma showed that treatment with sevoflurane was associated with reduced PCO2, improved pH, and reduction of peak airway pressures [143].

### 6.5. Myocardial Ischemia

Myocardial infarction often presents with significant IRI through many of the same mechanisms as cerebral ischemia [144]. IAs are frequently used as an anesthetic during interventional and surgical cardiac revascularization procedures, but their clinical benefit in this population remains uncertain. A 2007 meta-analysis by Landoni et al. showed that use of sevoflurane and desflurane are associated with reduced infarct size and improved mortality in cardiosurgical patients [145]. A more recent RCT of 867 patients comparing propofol-based TIVA to sevoflurane for elective bypass surgery showed reduced cardiac biomarker release and hospital length of stay for the sevoflurane group [146]. The majority of the current evidence for use of IAs in myocardial ischemia is comprised of preclinical data. A recent systematic review and meta-analysis of 37 preclinical trials included 313 controls and 536 subjects receiving sevoflurane in animal models of myocardial IRI [147]. Both preconditioning and postconditioning with sevoflurane were associated with significant reduction in infarct size. This analysis had several important limitations. Included studies had few data from large animals whose cardiovascular systems would more closely resemble that of humans, subjects lacked cardiovascular risk factors, and there was significant heterogeneity among studies. Given the sparsity and poor generalizability of existing data, further studies are needed to clarify the role of IAs in treatment of myocardial ischemia.

### 6.6. ICU Sedation

Critically ill patients often require sedation for management of agitation, mechanical ventilation, airway protection, or diagnostic or therapeutic procedures performed at bedside. While IV anesthetics are the mainstay of ICU sedation, IAs offer a short-acting, easily titratable alternative. A meta-analysis by Mesnil et al. of 523 patients undergoing long-term sedation in the ICU showed reduced time to extubation following cessation of sedation with sevoflurane versus sedation with either propofol or midazolam [148]. A separate meta-analysis by Kim et al. of 1027 patients showed that ICU sedation with sevoflurane not only reduced extubation times, but was also associated with lower troponin levels [149]. While long-term outcomes were not different in these analyses, a retrospective analysis of 369 surgical ICU patients showed that compared to sedation with propofol or midazolam, sedation with isoflurane is associated with more ventilator-free days, more hospital-free days, a lower risk of in-hospital mortality, and reduced risk of mortality at 1 year [150].

## 7. Practical Considerations

Despite their availability in most hospitals, the use of IAs outside the operating room continues to face challenges. Delivery of IAs requires specialized equipment and infrastructure [151]. Gas vaporizers are bulky and may be difficult to accommodate in space-constrained settings like the emergency department or ICU. Institutional and federal safety regulations dictate the need for waste gas scavenging and elimination devices. Pressure, flow, humidity, and temperature requirements of vaporizers may exceed the capabilities of existing infrastructure. Monitoring of anesthetic levels requires dedicated gas sensors and software on ventilators or patient monitors [152].

Cost is a significant consideration in effective implementation of IA protocols. There are few publicly available data on the cost effectiveness of IA. Vendor list prices and institutional acquisition prices are widely variable and often difficult to obtain. A cost-effectiveness analysis by Kampmier et al. concluded that sedation with propofol TIVA was less expensive than IA [153]. But this conclusion was reached based on aggregating data including differences in length of post-anesthesia care unit (PACU) stays, a consideration not applicable to patients sedated in critical care settings. Actual drug costs in this study were lower for the IA group. A separate single-center cost analysis of patients undergoing endoscopy procedures with TIVA versus IAs demonstrated lower drug costs with propofol compared to sevoflurane [154].

The environmental impact of IAs is another potential limitation to more widespread use. IAs account for nearly 3% of the healthcare-related carbon footprint of high-income countries [155]. The global warming potential of gases are primarily determined by their absorption spectra and atmospheric lifetimes. IAs act as greenhouse gases due to their strong absorption bands within the 8–14 μm infrared atmospheric window [156]. Under normal atmospheric conditions, this region is largely devoid of thermal radiation absorption. Accumulation of IAs increases trapping of outgoing atmospheric radiation. Halogenated hydrocarbons have relatively little reactivity with atmospheric hydroxyl (OH) radicals, the primary species responsible for clearing atmospheric pollutants. The atmospheric lifetimes of IAs varies significantly, estimated to be as little as 1 year for sevoflurane to over 100 years for N_2_O [157]. Of the modern IAs, desflurane is considered to have the greatest global warming potential, and thus may be less preferable for institutions looking to reduce their climate impact.

Of the various physiological complications associated with use of IAs, malignant hyperthermia (MH) is one of the most feared. MH is an inherited disorder of abnormal calcium release secondary to mutations of RYR1, CACNA1S, or STAC3 genes triggered by exposure to succinylcholine or halogenated anesthetics [158]. It presents as hypercarbia, hyperthermia, masseter spasm, rhabdomyolysis, and lactic acidosis. Recognition of MH may be challenging in anesthetized patients and requires continuous temperature and end-tidal CO_2_ monitoring. Additionally, first-line treatment involves both adequate resuscitation and administration of dantrolene, an infrequently used medication that is often not available outside the perioperative setting. Thus, safe use of IAs requires the ability to monitor for and treat this rare, but potentially lethal, complication.

## 8. Future Directions

Index-based depth of sedation monitoring is routinely used to titrate anesthetic doses to effect. At present, these devices use simplified electroencephalograph (EEG) and electromyogram (EMG) data to predict a patient’s depth of consciousness. Despite their use as a one-size-fits-all device for anesthetic monitoring, recent evidence suggests distinct differences in electroencephalograph EEG activity between sedation with IAs and IV anesthetics [159]. Modern advances in artificial intelligence (AI) algorithms are well-suited for real-time analysis of complex data such as EEG and EMG waveforms. Further development of such models could lead to true closed-loop sedation systems and automated data-driven titration of IAs to achieve desired sedation and hemodynamic goals.

Advances in genomic and proteomic technologies hold the potential to usher in a new age of precision anesthetic care and use of IAs tailored to individuals’ specific metabolic profiles [160]. While IAs are relatively insensitive to polymorphisms of metabolic enzymes, changes to tumor-related proteins may confer significantly different carcinogenic potential between individuals [161]. Individualized IA-regimens could be developed to help reduce inflammatory damage and cancer risk at the individual level.

One of the greatest challenges in synthesizing data surrounding IAs is the paucity of studies in non-operative patients. Most existing literature is limited by small sample size, high heterogeneity, and inconsistent definitions of endpoints. More well-powered prospective randomized control trials will be needed to fully realize the potential benefits of IAs beyond the operating room.

## Figures and Tables

**Table 1:** Minimum alveolar concentration (MAC) of commonly used inhalational anesthetics expressed as a percentage at 1 atmosphere absolute pressure (760 mmHg).

Agent	Minimum Alveolar Concentration (%)
Methoxyflurane	0.16
Halothane	0.75
Isoflurane	1.15
Enflurane	1.68
Sevoflurane	2.10
Desflurane	6
Nitrous Oxide	105
